# Detection of Prions in Wild Pigs (*Sus scrofa*) from Areas with Reported Chronic Wasting Disease Cases, United States

**DOI:** 10.3201/eid3101.240401

**Published:** 2025-01

**Authors:** Paulina Soto, Francisca Bravo-Risi, Rebeca Benavente, Tucker H. Stimming, Michael J. Bodenchuk, Patrick Whitley, Clint Turnage, Terry R. Spraker, Justin Greenlee, Glenn Telling, Jennifer Malmberg, Thomas Gidlewski, Tracy Nichols, Vienna R. Brown, Rodrigo Morales

**Affiliations:** The University of Texas Health Science Center at Houston, Texas, USA (P. Soto, F. Bravo-Risi, R. Benavente, T.H. Stimming, R. Morales); Centro Integrativo de Biologia y Quimica Aplicada, Universidad Bernardo O’Higgins, Santiago, Chile (P. Soto, F. Bravo-Risi, R. Morales); US Department of Agriculture, Fort Collins, Colorado, USA (M.J. Bodenchuk, P. Whitley, C. Turnage, J. Malmberg, T. Gidlewski, T. Nichols, V.R. Brown); Colorado State University, Fort Collins, Colorado, USA (T.R. Spraker, G. Telling); US Department of Agriculture, Ames, Iowa, USA (J. Greenlee)

**Keywords:** Chronic wasting disease, CWD, prions and related diseases, wild pigs, CWD ecology, zoonoses, interspecies prion transmission, Arkansas, Texas, United States

## Abstract

Using a prion amplification assay, we identified prions in tissues from wild pigs (*Sus scrofa*) living in areas of the United States with variable chronic wasting disease (CWD) epidemiology. Our findings indicate that scavenging swine could play a role in disseminating CWD and could therefore influence its epidemiology, geographic distribution, and interspecies spread.

Chronic wasting disease (CWD) is a prion disease of particular concern because of its uncontrolled contagious spread among various cervid species in North America (https://www.usgs.gov/media/images/distribution-chronic-wasting-disease-north-america-0), its recent discovery in Nordic countries (*1*), and its increasingly uncertain zoonotic potential (*2*). CWD is the only animal prion disease affecting captive as well as wild animals. Persistent shedding of prions by CWD-affected animals and resulting environmental contamination is considered a major route of transmission contributing to spread of the disease. Carcasses of CWD-affected animals represent relevant sources of prion infectivity to multiple animal species that can develop disease or act as vectors to spread infection to new locations.

Free-ranging deer are sympatric with multiple animal species, including some that act as predators, scavengers, or both. Experimental transmissions to study the potential for interspecies CWD transmissions have been attempted in raccoons, ferrets, cattle, sheep, and North American rodents (*3*–*7*). Potential interspecies CWD transmission has also been addressed using transgenic (Tg) mice expressing prion proteins (PrP) from relevant animal species (*8*). Although no reports of natural interspecies CWD transmissions have been documented, experimental studies strongly suggest the possibility for interspecies transmission in nature exists (*3*–*7*). Inoculation and serial passage studies reveal the potential of CWD prions to adapt to noncervid species, resulting in emergence of novel prion strains with unpredicted features (*9*–*11*).

Wild pigs (*Sus scrofa*), also called feral swine, are an invasive population comprising domestic swine, Eurasian wild boar, and hybrids of the 2 species (*12*). Wild pig populations have become established in the United States (Appendix Figure 1, panel A), enabled by their high rates of fecundity; omnivorous and opportunistic diet; and widespread, often human-mediated movement (*13*). Wild pigs scavenge carcasses on the landscape and have an intimate relationship with the soil because of their routine rooting and wallowing behaviors (*14*). CWD prions have been experimentally transmitted to domestic pigs by intracerebral and oral exposure routes (*15*), which is relevant because wild pigs coexist with cervids in CWD endemic areas and reportedly prey on fawns and scavenge deer carcasses. Considering the species overlap in many parts of the United States (Appendix Figure 1, panel B), we studied potential interactions between wild pigs and CWD prions.

## The Study

We screened pig tissues by using the protein misfolding cyclic amplification (PMCA) technique (Appendix). To screen for CWD and porcine-adapted CWD prions in wild pigs, we analyzed the sensitivity and specificity of PMCA to detect prions from different animal species. To evaluate, we first tested the PMCA efficiency of deer CWD prions in both deer and pig substrates. We found the in vitro replication of CWD prions was highly efficient in the deer substrate, and detectable even at high (10^−11^) dilutions in a first PMCA round (Appendix Figure 2). As a counterpart, we used a porcine-adapted PrP^Sc^ (scrapie isoform of infectious prion proteins) pool generated through >10 PMCA rounds in Tg002 substrate (Appendix Figure 3). That inoculum displayed limited PMCA seeding activity after 1 round, confirming the ability of our in vitro prion replication systems to discriminate between homologous (deer) and heterologous (porcine) PrP^Sc^ sources (Appendix Figure 2, panel A).

The specificity of our cervid and porcine PMCA systems was further confirmed when using the pig-derived PMCA substrate (Appendix Figure 2, panel B). Specifically, although we detected no amplification in the system when using the CWD seeds, the porcine-adapted PMCA product displayed great amplification. Overall, those results demonstrated that PMCA can be used to identify prions from deer and porcine sources, provide species specificity, and potentially enable tracking of the source of the infectious agent in tissues collected from wild pigs.

**Figure 1:**
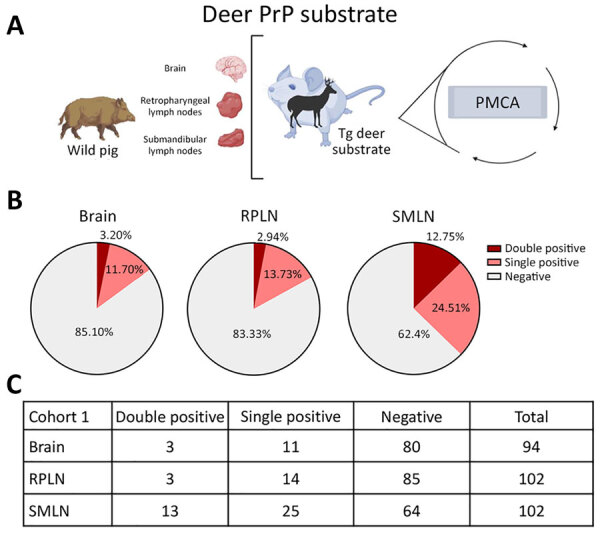
Prion detection using cervid-PMCA in wild pig tissues originating from a CWD-endemic region of Arkansas, USA, in a study of prions in wild pigs (*Sus scrofa*) from areas with reported chronic wasting disease cases. A) Experimental strategy depicting tissues analyzed in animals from this cohort and PMCA settings. B) Graphs representing percentage of detection per tissue. C) Details on the prion detection data displayed in panel B. CWD, chronic wasting disease; PMCA, protein misfolding cyclic amplification; PrP, prion proteins; RPLN, retropharyngeal lymph nodes; SMLN, submandibular lymph nodes; Tg, transgenic.

We then investigated the interaction between wild pigs and CWD prions in tissues from animals trapped in areas with different CWD epidemiology. The first cohort of pigs (cohort 1) included animals trapped in Newton and Searcy Counties, Arkansas, USA, where CWD is endemic in free-ranging white-tailed deer (*Odocoileus virginianus*) and elk (*Cervus elaphus canadensis*) (https://www.agfc.com/hunting/deer/chronic-wasting-disease/cwd-in-arkansas). The tissues considered for this screening included retropharyngeal lymph nodes (RPLN) and submandibular lymph nodes (SMLN) because those sites have been described to replicate prions shortly after infection in multiple animal species (Appendix). We also tested brainstem samples from the same animals. The first part of the screening involved the use of deer PrP substrate to assess for the potential exposure of wild pigs to CWD prions. We analyzed all samples in duplicate. We were able to detect CWD seeding activity in several wild pig lymphoid tissues and a higher proportion in the SMLN (37.26%) compared with RPLN (16.67%) (Figure 1). As expected, brains displayed a lower detection; just 14.90% of the specimens provided positive signals (Figure 1). We considered a tissue as positive for CWD prions if we noted positive signals in >1 of the 2 replicates. Along that line, a fraction (12.75%) of PMCA-positive SMLN tissues provided positive PMCA results in both replicates, suggesting a higher concentration of CWD prions in SMLN than RPLN or brains (≈3%) (Figure 1). Of note, the CWD infectivity titers in wild pig tissues appeared to be limited because just a fraction of Tg1536 mice (expressing the deer prion protein) inoculated with selected specimens displayed subclinical prion infection (Appendix Table 1, Figure 4).

**Figure 2:**
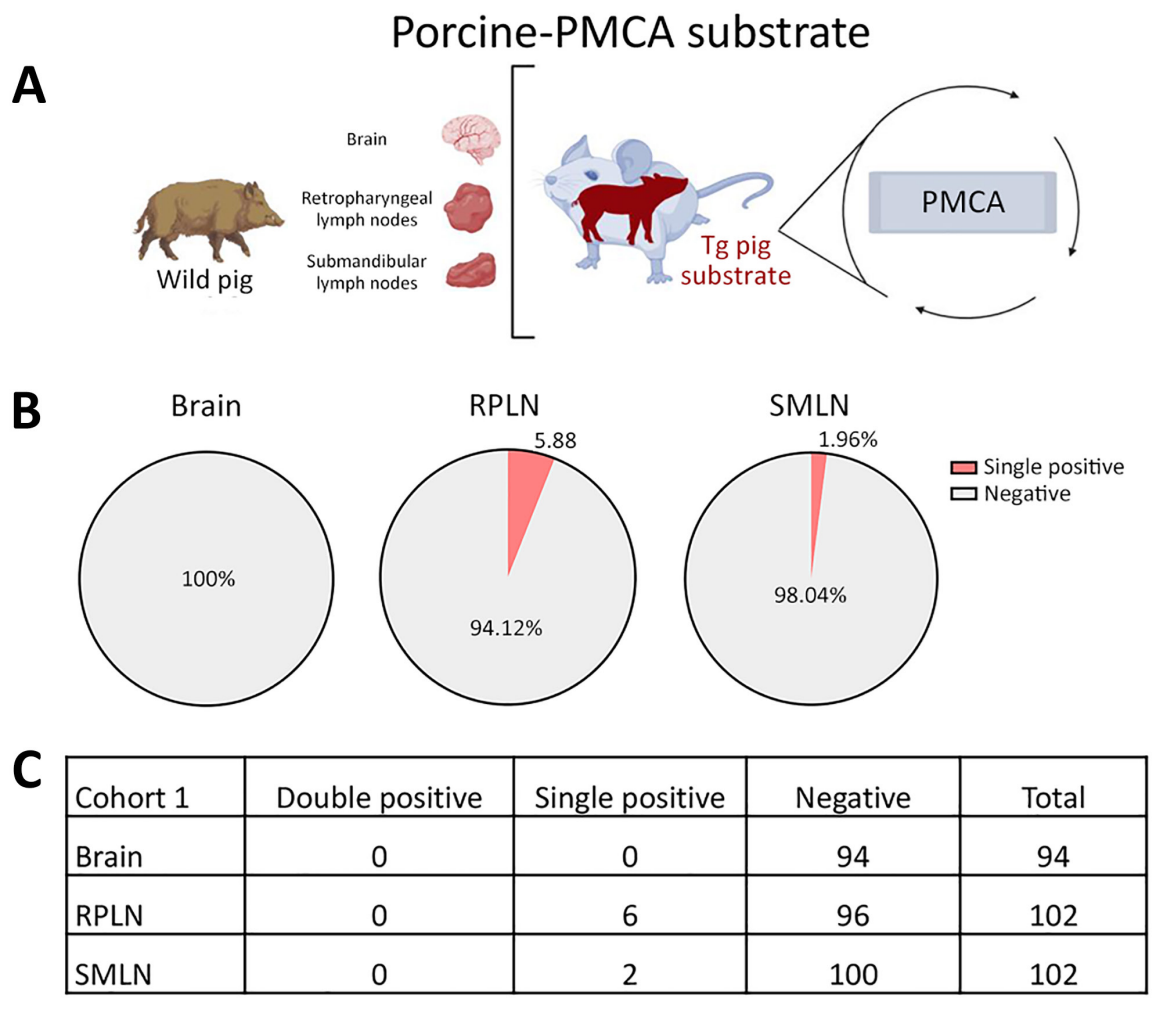
Prion detection using porcine-PMCA in wild pig tissues originating from a CWD-endemic region of Arkansas, USA, in a study of prions in wild pigs (*Sus scrofa*) from areas with reported chronic wasting disease cases. A) Experimental strategy depicting tissues analyzed in animals from this cohort and PMCA settings. B) Graphs representing percentage of detection per tissue. C) Details on the prion detection data displayed in panel B. PMCA, protein misfolding cyclic amplification; PrP, prion proteins; RPLN, retropharyngeal lymph nodes; SMLN, submandibular lymph nodes; Tg, transgenic.

Screening of the same tissues using porcine-PMCA substrate provided a considerably lower number of positive results (Figure 2). Although 5.88% of wild pigs were porcine-PMCA positive at RPLNs, only 1.96% were positive at SMLNs. Of note, none of the tissues that tested PMCA-positive using the porcine substrate provided positive signals in both replicates, suggesting low quantities of seeding-competent porcine prions (Figure 2). None of the brains from cohort 1 provided PMCA seeding activities when the porcine substrate was used, thus confirming those results (Figure 2). Moreover, bioassays in Tg002 mice expressing the porcine prion protein resulted in no transmission (Appendix Table 2).

**Figure 3:**
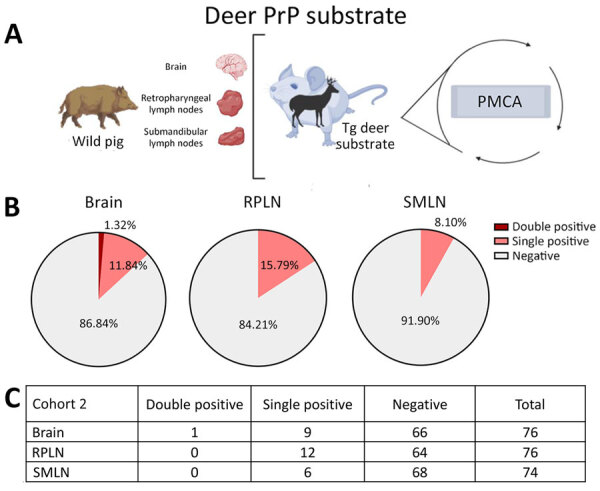
Prion detection using cervid-PMCA in wild pig tissues originating from a CWD-endemic region of Texas, USA, in a study of prions in wild pigs (*Sus scrofa*) from areas with reported chronic wasting disease cases. A) Experimental strategy depicting tissues analyzed in animals from this cohort and PMCA settings. B) Graphs representing percentage of detection per tissue. C) Details on the prion detection data displayed in panel B. CWD, chronic wasting disease; PMCA, protein misfolding cyclic amplification; PrP, prion proteins; RPLN, retropharyngeal lymph nodes; SMLN, submandibular lymph nodes; Tg, transgenic.

**Figure 4:**
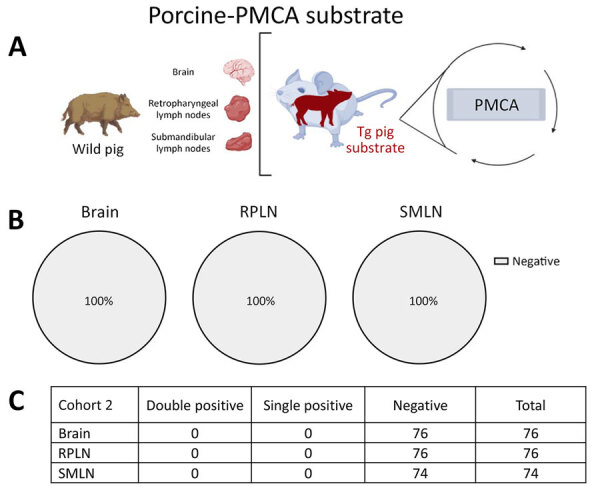
Prion detection using porcine-PMCA in wild pig tissues originating from a CWD-endemic region of Texas, USA, in a study of prions in wild pigs (*Sus scrofa*) from areas with reported chronic wasting disease cases. A) Experimental strategy depicting tissues analyzed in animals from this cohort and PMCA settings. B) Graphs representing percentage of detection per tissue. Grey samples are representative of no detection. C) Details on the prion detection data displayed in panel B. CWD, chronic wasting disease; PMCA, protein misfolding cyclic amplification; PrP, prion proteins; RPLN, retropharyngeal lymph nodes; SMLN, submandibular lymph nodes; Tg, transgenic.

Next, we conducted a screening on a second cohort of animals (cohort 2) collected in Texas, USA. Pigs from cohort 2 were retrieved from counties where either no or low-prevalence CWD had been reported in wild deer. The cervid-adapted PMCA analysis revealed that 15.79% of animals tested positive for CWD prions in RPLNs (Figure 3). Brains (13.16%) and SMLN (8.10%) were also positive, albeit in a lower percentage than for RPLN (Figure 3). Regardless, the overall proportion of PMCA-positive tissues was considerably lower compared with those found for cohort 1, in line with the low prevalence of CWD in free-ranging cervids in the Texas study region. In agreement with the presumably lower exposure of CWD prions for pigs from cohort 2, none of the tissues provided PMCA-positive signals when evaluated in the porcine PMCA system (Figure 4).

## Conclusions

In summary, results from this study showed that wild pigs are exposed to cervid prions, although the pigs seem to display some resistance to infection via natural exposure. Future studies should address the susceptibility of this invasive animal species to the multiple prion strains circulating in the environment. Nonetheless, identification of CWD prions in wild pig tissues indicated the potential for pigs to move prions across the landscape, which may, in turn, influence the epidemiology and geographic spread of CWD. 

Appendix Additional information on detection of prions in wild pigs (*Sus scrofa*) from areas with reported chronic wasting disease cases.
