# Supplementary material for: Detection of Prions in Wild Pigs (Sus scrofa) from Areas with Reported Chronic Wasting Disease Cases, United States
Source: Emerg Infect Dis. 2025 Jan;31(1):168–73. doi: 10.3201/eid3101.240401 (PMC11682794; doi:10.3201/eid3101.240401)
Supplement: Appendix — Additional information on detection of prions in wild pigs (Sus scrofa) from areas with reported chronic wasting disease cases. [file 24-0401-Techapp-s1.pdf]

Article DOI: https://doi.org/10.3201/eid3101.240401

EID cannot ensure accessibility for supplementary materials supplied by authors. Readers who have difficulty accessing supplementary content should contact the authors for assistance.

Detection of Prions in Wild Pigs (*Sus scrofa*) from Areas with Reported Chronic Wasting Disease Cases, United States

Appendix

Prion diseases are fatal neurodegenerative disorders affecting several mammalian species including humans and livestock (*1*). Chronic wasting disease (CWD) is of particular concern because of its uncontrolled contagious spread among various cervid species in North America (https://www.usgs.gov/media/images/distribution-chronic-wasting-disease-north-america-0), its recent discovery in Nordic countries (*2*), and its increasingly uncertain zoonotic potential (*3*). Importantly, CWD is the only animal prion disease affecting captive as well as wild animals.

Persistent shedding by CWD-affected animals and resulting environmental contamination is considered a significant route of transmission which contributes to the spreading of the disease. CWD prions present in feces, urine, saliva, and tissues released after fawning are considered to be significant for environmental contamination (*4*,*5*). Carcasses of CWD-affected animals also represent relevant sources of prion infectivity to multiple animal species that may either develop disease or else act as vectors to spread infection to remote locations. Prions are resistant to denaturation by multiple environmental conditions (*4*,*6*,*7*). Disease transmission is also thought to be influenced by the ability of prions to bind to soils and other natural and anthropogenic materials (*8*–*13*).

Free-ranging deer are sympatric with multiple animal species, including some that may act as predators and/or scavengers. Experimental transmissions to study the potential for inter-species CWD transmissions have been attempted in raccoons (*14*), ferrets (*15*), cattle (*16*), sheep (*17*) and North American rodents (*18*). This has also been addressed using transgenic (Tg) mice expressing the prion proteins (PrP) from relevant animal species (*19*). Although no reports of natural inter-species CWD transmissions have been documented, the possibility of these events

to occur in nature exist based in the aforementioned experimental studies. Inoculation and serial passage studies reveal the potential of CWD prions to adapt to non-cervid species, resulting in the emergence of novel prion strains with unpredicted features (*20–22*).

Wild pigs, or feral swine, are an invasive population comprising domestic swine, Eurasian wild boar, and hybrids of the two (*23*). The establishment of wild pig populations in the United States (Appendix Figure 1, panel A) is facilitated by their high rate of fecundity, omnivorous and opportunistic diet, and widespread, often human-mediated movement (*24*). Wild pigs scavenge carcasses on the landscape and have an intimate relationship with the soil given their routine rooting and wallowing behaviors (*25*). CWD prions have been experimentally transmitted to domestic pigs by intracerebral and oral routes of exposure (*26*). This is relevant since wild pigs co-exist with cervids in CWD endemic areas and reportedly prey on fawns and scavenge deer carcasses. In light of this overlap in many parts of the United States (Appendix Figure 1, panel B), we studied potential interactions between wild pigs and CWD prions.

Materials And Methods

Samples

Brainstem (Cohort 1), brain (Cohort 2), retropharyngeal lymph nodes (RPLN) (both Cohorts), and submandibular lymph nodes (SMLN) (both Cohorts) were collected from two wild pig cohorts. Samples from the first cohort (n = 102 for RPLN and SMLN, and n = 94 for brainstem) were collected from Newton and Searcy Counties, Arkansas, where CWD is endemic in free-ranging white-tailed deer (*Odocoileus virginianus*) and elk (*Cervus canadensis*) (CWD in Arkansas (agfc.com)). Samples from the second cohort (n = 76 for brain and RPLN, and n = 74 for SMLN) were collected from Hartley, Oldham, and Potter counties in Texas where CWD have either not yet been detected or is present at low prevalence in free-ranging populations at the time of collection (Distribution of Chronic Wasting Disease in North America | U.S. Geological Survey (usgs.gov)). All samples were collected by USDA Wildlife Services personnel from adult (characterized as being reproductively active) male and female wild pigs. Brains were homogenized at a concentration of 10% w/v (weight/volume) in phosphate-buffered saline (PBS) (Hyclone PBS, GE Healthcare Life Sciences), and supplemented with a protease inhibitor cocktail without EDTA (Roche). Brains were homogenized using 2 mL volume Precellys® Soft

Tissue Homogenizer tubes (Bertin Corp) using program #3 in a Precellys® 24 homogenizer. RPLN and SMLN tissues were prepared at a concentration of 20% w/v in the same buffer and homogenized in hard tissue homogenizing tubes using program #5 in the Precellys® 24 homogenizer. We used one homogenization cycle for brains and three homogenization cycles for lymph nodes.

Animal Models

We used Tg1536 (*27*) and Tg002 (*26*) mice in this study, transgenic animals expressing the deer and porcine versions of the prion protein (PrP), respectively.

PMCA Procedure

Homozygous Tg1536 and heterozygous Tg002 mice were used to prepare PMCA substrates. The PMCA assay was conducted as previously described (*12*,*13*,*28*) with minor modifications. These include supplementations in PMCA substrates as follows: Tg1536 homogenates were supplemented with 5 mM EDTA and digitonin 0.025% (TgDeer substrate), and Tg002 homogenates were supplemented with 5 mM EDTA and 0.05% of digitonin (TgPig substrate). Each sample was tested in duplicate by different investigators. PMCA statuses of samples were evaluated in a third PMCA round.

Pre-PMCA Processing of Wild Pig Tissues

Brain and lymph node samples were processed by incubating 200 µL of the tissue homogenate with an equal volume of either 10% or 20% sarkosyl prepared with ultrapure water. Mixtures were incubated for 1 hour (h) at room temperature and centrifuged at 100,000 x *g* and 4 °C for 1 h. Supernatants were discarded and pellets were washed with 400 µL PBS supplemented with a protease inhibitor cocktail lacking EDTA (Roche). Samples were centrifuged again at 100,000 x *g* for 30 min at 4°C. Pellets were resuspended directly in the PMCA substrate.

Bioassays

Male and female hemizygous Tg1536 and Tg002 mice were used for prion infectivity bioassays (46.2% females). All animal experimentations were performed following NIH guidelines and approved by the local Animal Welfare Committee (protocol number 20-0065). Animal injections, monitoring and sacrificing are similar as described in (*13*,*29*). In this experiment, each mouse was stereotaxically injected with 10 µL of a 10% (brain) or 20% (lymph nodes) w/v wild pig tissue homogenate in the right hippocampus. The potential presence of PrP^{Sc}

in brain samples was evaluated by western blotting. Brains displaying negative PrP^{Sc} signals were further analyzed by three PMCA rounds.

Results

In vitro Amplification of CWD Prions in Cervid and Porcine Substrates

To screen for CWD and porcine-adapted CWD prions in wild pigs, we analyzed the sensitivity and specificity of PMCA to detect prions from these different animals. We have extensively published PMCA analyses for CWD prions in different contexts, including diagnosis, environmental contamination and disease mechanisms (*12*,*13*,*30–34*). However, we have not explored whether our deer PMCA protocol is able to replicate porcine-adapted prions. Along the same line, it is currently unknown if CWD prions are able to induce the misfolding of the pig prion protein in vitro. To evaluate this, we first tested the PMCA efficiency of CWD prions in both deer and pig substrates. CWD prions were obtained from the brain extract of a terminally ill, experimentally infected white-tailed deer that was intra-cerebrally infected. As shown in Appendix Figure 2, the in vitro replication of CWD prions was highly efficient in the deer substrate, being detectable even at high dilutions (*10−11*) in a first PMCA round. As counterpart, we used a porcine-adapted PrP^{Sc} pool generated through >10 PMCA rounds in Tg002 substrate. The original inoculum for this PMCA product was the brain of an experimentally scrapie-infected domestic pig. The porcine-adapted PrP^{Sc} displayed high quantities of PK-resistant PrP^{Sc} as evaluated by western blot (Appendix Figure 3). As shown in Appendix Figure 2, panel A, this inoculum displayed limited PMCA seeding activity after 1 round, confirming the ability of our in vitro prion replication systems to discriminate between homologous (deer) and heterologous (porcine) PrP sources.

The specificity of our cervid and porcine PMCA systems was further confirmed when using the pig-derived PMCA substrate (Appendix Figure 2, panel B). Specifically, while no amplification was detected in this system when using the CWD seeds, the porcine-adapted PMCA product displayed great amplification. Overall, the results presented in Supplementary Figure 2 demonstrate that PMCA is able to identify prions from deer and porcine sources, while providing with species-specificity and the ability of potentially tracking the source of the infectious agent in tissues collected from wild pigs.

Screening of Cohort 1 of Wild Pigs by PMCA using Cervid and Porcine Substrates

The interaction between wild pigs and CWD prions was studied in tissues from animals trapped in areas with different CWD epidemiology. The first cohort of pigs (Cohort 1) included animals trapped in Newton and Searcy Counties, Arkansas, where CWD is endemic in free-ranging white-tailed deer and elk (Arkansas Game and Fish Commission, 2023). The tissues considered for this screening included lymph nodes as they have been described to replicate prions shortly after infection in multiple animal species (*35–37*). Specifically, retropharyngeal (RPLN) and submandibular (SMLN) lymph nodes were selected considering their relevance in the diagnosis of CWD in deer (*33*,*35*). In addition, brainstem samples from the same animals were also selected to assess for potential neuroinvasion. The first part of this screening involved the use of deer PrP substrate to assess for the potential exposure of wild pigs to CWD prions. All samples were analyzed in duplicate and results are listed in Figure 1. We were able to detect CWD seeding activity in several wild pig lymphoid tissues, with a higher proportion in the SMLN 37.26% animals compared to RPLN (16.67%) (Figure 1). As expected, brains displayed a lower detection considering that just 14.90% of the specimens provided positive signals (Figure 1). We considered a tissue as positive for CWD prions if they provided positive signals in at least one of the two replicates. Along this line, a fraction of the PMCA positive SMLN tissues (12.75%) provided positive PMCA results in both replicates, suggesting a higher presence of CWD prions on them compared with RPLN and brains (≈3%) (Figure 1).

The screening of the same tissues using porcine-PMCA substrate provided a considerably lower number of positive results (Figure 2). While 5.88% of wild pigs were porcine-PMCA positive at RPLNs, only 1.96% were positive at the SMLN. Importantly, none of the PMCA-positive tissues using the porcine substrate provided positive signals in both replicates, suggesting low quantities of seeding-competent porcine prions (Figure 2). Confirming these results, none of the brains from this cohort provided PMCA seeding activities when the porcine substrate was used (Figure 2).

Overall, the results collected from this first cohort suggests that wild pigs living in CWD endemic areas are indeed exposed to CWD prions. Our data also suggest that the replication of cervid prions in pigs is limited.

Bioassays of Selected Brain and Lymph Node Wild Pig Tissues in Tg1536 and Tg002 Mice

It is known that PMCA can detect prions at sub-infectious concentrations (*13*,*38*,*39*). Considering this, the data presented above cannot be interpreted as surrogates of prion infectivity. To address this issue, we injected wild pig tissues from the study above (Cohort I) in Tg1536 and Tg002 mice. Tissues were selected considering at least two of the following criteria: i) positive PMCA detection in cervid substrate in both replicates, ii) positive PMCA detection in more than one tissue in either cervid or porcine substrates, iii) positive PMCA detection in at least one tissue in both cervid and porcine substrates. These criteria provided n = 3 SMLN, n = 1 RPLN and n = 2 brain tissues selected for the bioassay. A brain extract from a prion-free domestic pig (ID000000) was used as negative control, while the brain of an experimentally CWD-infected, terminally ill white-tailed deer and the PMCA-adapted porcine PrP^{Sc} described in Appendix Figure 3 were used as positive controls.

As shown in Appendix Table 1, none of the Tg1536 mice treated with the wild pig tissues resulted in clinical prion disease after 600 days post-inoculation. Pre-clinical prion infection in these mice was evaluated by western blot and three PMCA rounds in homologous (Tg1536) substrate. Western blot data did not provide evidence of prion transmission in any of the mice included in this study. However, the brains from five mice were positive in PMCA (Appendix Figure 4). Specifically, all mice identified for subclinical prion infection were treated with two different SMLN samples. The presence of pre-clinical prion infection in a low fraction of animals is strongly suggestive of low prion infectivity titers in these tissues. Bioassays in Tg002 mice did not result in transmission as none of these mice developed clinical or pre-clinical infection (Figure 4; Appendix Table 2).

In summary, the bioassay results demonstrate that prion detection in wild pig tissues by PMCA are present at sub-infectious levels. The presence of pre-clinical, prion infected Tg1536 mice in this bioassay further confirms that wild pigs are naturally exposed to CWD prions. However, the absence of transmission in Tg002 mice suggest that porcine-adapted CWD prions exist at concentrations that are not relevant for disease transmission in wild pigs.

Screening of Cohort 2 of Wild Pigs by PMCA using Cervid and Porcine Substrates

We conducted a screening on a second cohort of animals (Cohort 2) collected in Texas. Wild pigs from Cohort 2 were retrieved from counties where either no or low-prevalence CWD

had been reported in wild deer. The PMCA screening of RPLN, SMLN and brain tissues from these animals was performed using white-tailed deer and porcine substrates, as described above. The cervid-adapted PMCA analysis revealed that 15.79% of animals tested positive for CWD prions in RPLNs (Figure 3). Brains (13.16%) and SMLN (8.10%) were also positive, albeit in a lower proportion compared with RPLN (Figure 3). Regardless, the overall proportion of PMCA positive tissues was considerably lower compared to that found for Cohort I, in line with the low prevalence of CWD in free-ranging cervids in this study region. It is important to note that all tissues in Cohort 2, with the exception of a single brain specimen, provided positive-PMCA signals in only one of the two replicates included in this study (Figure 3). In agreement with the presumably lower exposure of CWD prions for pigs from Cohort 2, none of the tissues provided PMCA-positive signals when evaluated in the porcine PMCA system (Figure 4).

Discussion

The mechanisms contributing to the natural spread of CWD have been extensively studied (*4*,*5*,*40*). These research efforts highlight the gaps in our understanding of how CWD spreads. In this context, the host range of CWD under natural ecologic circumstances is an important consideration. Previous knowledge on this topic comes mostly from experimental studies where the CWD agent has been administered to multiple animal species that are sympatric with cervids. These experiments include CWD exposure to racoons (*14*), ferrets (*41*,*42*), cattle (*43*), sheep (*17*), pigs (*26*) and rodents (*18*). Although several of these experiments demonstrate that CWD can be transmitted and adapted to multiple animal species, most of them have been performed via intra-cerebral administrations of brain extracts containing high concentrations prions. Needless to say, these scenarios do not represent naturally occurring events. At present, no cases of naturally occurring inter-species CWD transmission have been reported. Along this line, the natural host range of CWD is still unknown. One species that is potentially in contact with CWD infected animals in natural settings is the wild pig. These animals prey on fawns and scavenge on cervid carcasses. Worrisomely, a relatively recent experimental study described that domestic pigs intracerebrally or orally challenged with CWD prions are susceptible (*26*). This information prompted us to investigate natural exposure of wild pigs to CWD on the landscape.

To analyze a large number of wild pigs, we used the PMCA technique that is able to replicate prions in an accelerated manner (*28*). Considering that PMCA shows specificity for prions from different animal species (*28*,*44*,*45*) we used both deer and porcine substrates and seeded PMCA reactions with prions originating from both pigs and deer. Interestingly, the PMCA variants used in this study demonstrated species specificities that were advantageous for our screening. Specifically, we observed strong in vitro prion replication when homologous inoculum/substrate combinations were used, and low efficiencies when heterologous pairings were tested (Appendix Figure 2). Using these PMCA settings, we evaluated tissues from wild pigs collected from geographic regions with variable CWD status. The detection of CWD prions by PMCA suggests that wild pigs are indeed naturally exposed to the infectious agent on the landscape. We found that the percentage of positive PMCA reactions correlated with the CWD status of each geographic region. In some cases, CWD prion detection in wild pig tissues exceeded the estimated prevalence for CWD in free-ranging cervids within the study region (over 10% in some cases) (*33*). One explanation for these results is that the pigs collected in these restricted areas could be exposed to specific, common foci of CWD. Regardless, the multiple positive and negative controls used in this study supported the accuracy of the results. It is important to note that the samples tested in this study were pre-treated (concentrated for prions) before PMCA testing, unlike other studies performed by us using white-tailed deer samples (*33*). The latter can also explain the success of detection in the current study. In addition, it is important to mention that each sample was tested in duplicate, by two different investigators. The PMCA data in both cases was interpreted in the same way: as positive and negative. Due to the ultrasensitive nature of PMCA, there is an intrinsic variability in this technique. Considering this, PMCA data cannot be interpreted based on the intensity of the bands obtained via western blot. We found that positive and negative data was randomly distributed across the different manipulators, discarding any potential bias in the interpretation of the results. According to our experience, the appearance of PMCA signals in single replicates is common in samples containing low prion loads. It has been previously described that PMCA can detect up to a single particle of the infectious agent (*38*). Consequently, we believe that a dissimilar number of PMCA active molecules may be taken in different aliquots, explaining the presence of positive signals in a single replicate. Importantly, the CWD prions identified in wild pig tissues were present in low quantities as they induced detectable PMCA seeding activity in just a

fraction of the inoculated Tg1536 mice. In summary, our PMCA and bioassay data importantly suggest that wild pigs are indeed exposed to CWD prions in natural conditions.

The fact that wild pigs interact with CWD prions opens up two different potential scenarios: wild pigs may be either i) getting infected by CWD prions, or ii) moving prions across the landscape without being infected. We assumed that wild pigs exposed to CWD prions through consumption of infected cervids could have detectable prions in their tissues without being actively infected. We investigated these potential scenarios through a combination of in vitro and in vivo tests. Specifically, PMCA was used to determine if prions could be detected in tissues from naturally exposed wild pigs. A mouse bioassay was then used to determine if PMCA-positive tissues were able to transmit the disease. We found that pig tissues from the CWD-endemic Arkansas study region were more likely to be positive by PMCA with a cervid substrate compared to a porcine substrate. This suggests a robust species barrier between CWD prions and wild pigs in natural conditions. Bioassay of wild pig tissues from Arkansas provided further evidence of this as transmission was not observed in any challenged Tg002 mice. We expected less detection of CWD prions by PMCA in wild pig tissues originating from our study region in Texas, where CWD is either not yet detected or present at low prevalence. This was actually the case, as no in vitro seeding activity was observed for any tissue derived from Texas animals when tested in the porcine-PMCA assay. Importantly, the in vivo and in vitro derived data using the porcine prion protein expressing mice demonstrate that wild pigs are not readily infected by CWD prions through natural exposure.

Although the findings presented in this article are significant, there are obvious limitations that should be addressed in future studies. One of them involves the limited geographic sampling of wild pigs. CWD has been identified in numerous U.S. states and Canadian provinces, affecting a variety of cervid species replicating numerous prion strains. Prion strains are known to display different host ranges under experimental conditions (*46*). Along this line, the susceptibility of wild pigs to naturally occurring CWD prion strains in experimental and natural scenarios should be a priority of future research efforts. A second limitation of our study includes the limited number of samples tested in bioassays. As mentioned above, wild pigs could be exposed to different prion strains and whether this was the case in our cohorts is unknown. Bioassays using a larger number of wild pig tissues should be conducted in future studies to further assess the potential inter-species transmission of CWD in natural

scenarios. A third limitation is that we were unable to present a true negative control, which is largely due to the rapid spread of CWD across the U.S. and the changing status of this disease in many states and counties. All counties from which we obtained samples have either had recent CWD detections or border a county with recent CWD detections, indicating that the presence of CWD is likely even if it has not yet been detected. In other words, samples were not available from a region in which we have high confidence that CWD is truly absent. We therefore opted to compare across two areas with different CWD status: relatively high prevalence versus absent to low prevalence. The later statements are supported in the fact that the CWD status of Oldham County (Texas) has recently changed due to a new detection. Moreover, the containment zone in the Texas area has been expanded into Potter County, since this county borders a county with recent detections. Based on this, we did not aim to segregate the samples between Texas counties and instead opted to treat this as a region with recent detections and relatively low prevalence for comparison to Arkansas, which is a state with endemic CWD at a relatively high prevalence. Relevant to the latter statements, CWD status is defined by animals tested by IHC and/or ELISA. A study published by our group last year unveiled, through PMCA testing, CWD positive animals in Texas counties with no previously reported cases (*33*). This data was obtained from deer's RPLN previously tested as CWD non-detect by IHC and ELISA. As mentioned above, these results support the idea that prion amplification assays are more sensitive than the currently approved methods when the same sample type is interrogated. We recognize that the lack of sampling in a truly negative CWD area is a limitation of this study, as is the case for many wildlife studies that rely on opportunistic sampling and data. Nonetheless, we feel that the comparison is informative, and set the basis for future studies assessing the interaction between CWD and wild pigs.

Despite the evidence presented here, the interaction between pigs and CWD prions may still have implications for the ecology of this infectious agent. As mentioned, different animal species display variable susceptibility to CWD prions under experimental conditions. This suggests that co-existing animals in contact with prions, such as predators and scavengers, might not get infected but act as mechanical spreaders of infectious particles. This has been suggested for coyotes and crows that release prions after passing them through their digestive tracts (*47*,*48*), although these events could result in a substantial reduction of prion infectivity titers as described for mountain lions (*49*). Wild pigs could also disseminate prions after passing them

through their digestive tracts or after the ingestion of contaminated environmental components (e.g., soils, plants, etc.). In North America, the average home ranges of feral pigs have been estimated at 1.1 - 5.32 Km^2 (*50–53*). Larger home-range sizes are reported for feral pigs in harsher climates with less food availability. This home-range size is sufficient to warrant concern for spread of CWD beyond containment zones or beyond the borders of CWD-positive management units in states where managers are working to mitigate CWD spread. Although spread by cervids is likely of greater epidemiologic relevance, feral pigs could complicate expensive and labor-intensive efforts to contain CWD.

This study did not attempt to directly address the potential for CWD to adapt to wild pigs via serial passage through oral exposure. Wild pigs often engage in cannibalism and con-specific scavenging, which presents a unique risk for prion disease transmission. Wild pigs may engage in cannibalism when interacting with the corpses of their conspecifics, and this has been described as a potential contributing factor to the transmission of African swine fever (*54*). Considering this evidence, the natural serial passage of infectious prions between pigs cannot be discarded. We caution that wild pig consumption of porcine tissue containing CWD prions could result in adaptation to a novel host, and emergence of a prion strain of unknown host range.

In summary, our results show that wild pigs are exposed to cervid prions, although they seem to display some resistance to infection via natural exposure. The identification of CWD prions in wild pig tissues indicates the potential for pigs to move prions across the landscape, which may in turn influence the epidemiology and geographic spread of CWD. Future studies should address the susceptibility of this invasive animal species to the multiple prion strains circulating in the environment.

References

1. Collinge J. Prion diseases of humans and animals: their causes and molecular basis. Annu Rev Neurosci. 2001;24:519–50. PubMed https://doi.org/10.1146/annurev.neuro.24.1.519

2. Benestad SL, Mitchell G, Simmons M, Ytrehus B, Vikøren T. First case of chronic wasting disease in Europe in a Norwegian free-ranging reindeer. Vet Res. 2016;47:88. PubMed https://doi.org/10.1186/s13567-016-0375-4

3. Tranulis MA, Tryland M. The zoonotic potential of chronic wasting disease—a review. Foods. 2023;12:824. PubMed https://doi.org/10.3390/foods12040824

4. Zabel M, Ortega A. The ecology of prions. Microbiol Mol Biol Rev. 2017;81:e00001-17. PubMed https://doi.org/10.1128/MMBR.00001-17

5. Bartelt-Hunt SL, Bartz JC. Behavior of prions in the environment: implications for prion biology. PLoS Pathog. 2013;9:e1003113. PubMed https://doi.org/10.1371/journal.ppat.1003113

6. Prusiner SB. Prions. Proc Natl Acad Sci U S A. 1998;95:13363–83. PubMed https://doi.org/10.1073/pnas.95.23.13363

7. Yuan Q, Telling G, Bartelt-Hunt SL, Bartz JC. Dehydration of prions on environmentally relevant surfaces protects them from inactivation by freezing and thawing. J Virol. 2018;92:e02191-17. PubMed https://doi.org/10.1128/JVI.02191-17

8. Smith CB, Booth CJ, Pedersen JA. Fate of prions in soil: a review. J Environ Qual. 2011;40:449–61. PubMed https://doi.org/10.2134/jeq2010.0412

9. Pritzkow S, Morales R, Lyon A, Concha-Marambio L, Urayama A, Soto C. Efficient prion disease transmission through common environmental materials. J Biol Chem. 2018;293:3363–73. PubMed https://doi.org/10.1074/jbc.M117.810747

10. Pritzkow S, Morales R, Moda F, Khan U, Telling GC, Hoover E, et al. Grass plants bind, retain, uptake, and transport infectious prions. Cell Rep. 2015;11:1168–75. PubMed https://doi.org/10.1016/j.celrep.2015.04.036

11. Carlson CM, Thomas S, Keating MW, Soto P, Gibbs NM, Chang H, et al. Plants as vectors for environmental prion transmission. iScience. 2023;26:108428. PubMed https://doi.org/10.1016/j.isci.2023.108428

12. Soto P, Bravo-Risi F, Benavente R, Lichtenberg S, Lockwood M, Reed JH, et al. Identification of chronic wasting disease prions in decaying tongue tissues from exhumed white-tailed deer. mSphere. 2023;8:e00272-23. https://doi.org/10.1128/msphere.00272-23

13. Soto P, Bravo-Risi F, Kramm C, Gamez N, Benavente R, Bonilla DL, et al. Nasal bots carry relevant titers of CWD prions in naturally infected white-tailed deer. EMBO Rep. 2024;25:334–50. https://doi.org/10.1038/s44319-023-00003-7

14. Moore SJ, Smith JD, Richt JA, Greenlee JJ. Raccoons accumulate PrP^{Sc} after intracranial inoculation of the agents of chronic wasting disease or transmissible mink encephalopathy but not atypical scrapie. J Vet Diagn Invest. 2019;31:200–9. PubMed https://doi.org/10.1177/1040638718825290

15. Sigurdson CJ, Mathiason CK, Perrott MR, Eliason GA, Spraker TR, Glatzel M, et al. Experimental chronic wasting disease (CWD) in the ferret. J Comp Pathol. 2008;138:189–96. PubMed https://doi.org/10.1016/j.jcpa.2008.01.004

16. Greenlee JJ, Nicholson EM, Smith JD, Kunkle RA, Hamir AN. Susceptibility of cattle to the agent of chronic wasting disease from elk after intracranial inoculation. J Vet Diagn Invest. 2012;24:1087–93. PubMed https://doi.org/10.1177/1040638712461249

17. Cassmann ED, Moore SJ, Greenlee JJ. Experimental oronasal transmission of chronic wasting disease agent from white-tailed deer to Suffolk sheep. Emerg Infect Dis. 2021;27:3156–8. PubMed https://doi.org/10.3201/eid2712.204978

18. Heisey DM, Mickelsen NA, Schneider JR, Johnson CJ, Johnson CJ, Langenberg JA, et al. Chronic wasting disease (CWD) susceptibility of several North American rodents that are sympatric with cervid CWD epidemics. J Virol. 2010;84:210–5. PubMed https://doi.org/10.1128/JVI.00560-09

19. Herbst A, Wohlgemuth S, Yang J, Castle AR, Moreno DM, Otero A, et al. Susceptibility of beavers to chronic wasting disease. Biology (Basel). 2022;11:667. PubMed https://doi.org/10.3390/biology11050667

20. Morales R, Abid K, Soto C. The prion strain phenomenon: molecular basis and unprecedented features. Biochim Biophys Acta. 2007;1772:681–91. PubMed https://doi.org/10.1016/j.bbadis.2006.12.006

21. Morales R. Prion strains in mammals: different conformations leading to disease. PLoS Pathog. 2017;13:e1006323. PubMed https://doi.org/10.1371/journal.ppat.1006323

22. Otero A, Duque Velasquez C, McKenzie D, Aiken J. Emergence of CWD strains. Cell Tissue Res. 2023;392:135–48. PubMed https://doi.org/10.1007/s00441-022-03688-9

23. Smyser TJ, Tabak MA, Slootmaker C, Robeson MS II, Miller RS, Bosse M, et al. Mixed ancestry from wild and domestic lineages contributes to the rapid expansion of invasive feral swine. Mol Ecol. 2020;29:1103–19. PubMed https://doi.org/10.1111/mec.15392

24. Bevins SN, Pedersen K, Lutman MW, Gidlewski T, Deliberto TJ. Consequences associated with the recent range expansion of nonnative feral swine. Bioscience. 2014;64:291–9. https://doi.org/10.1093/biosci/biu015

25. Graves HB. Behavior and ecology of wild and feral swine (*Sus scrofa*). J Anim Sci. 1984;58:482–92. https://doi.org/10.2527/jas1984.582482x

26. Moore SJ, West Greenlee MH, Kondru N, Manne S, Smith JD, Kunkle RA, et al. Experimental transmission of the chronic wasting disease agent to swine after oral or intracranial inoculation. J Virol. 2017;91:e00926-17. PubMed https://doi.org/10.1128/JVI.00926-17

27. Browning SR, Mason GL, Seward T, Green M, Eliason GAJ, Mathiason C, et al. Transmission of prions from mule deer and elk with chronic wasting disease to transgenic mice expressing cervid PrP. J Virol. 2004;78:13345–50. PubMed https://doi.org/10.1128/JVI.78.23.13345-13350.2004

28. Morales R, Duran-Aniotz C, Diaz-Espinoza R, Camacho MV, Soto C. Protein misfolding cyclic amplification of infectious prions. Nat Protoc. 2012;7:1397–409. PubMed https://doi.org/10.1038/nprot.2012.067

29. Castilla J, Morales R, Saá P, Barria M, Gambetti P, Soto C. Cell-free propagation of prion strains. EMBO J. 2008;27:2557–66. PubMed https://doi.org/10.1038/emboj.2008.181

30. Kramm C, Pritzkow S, Lyon A, Nichols T, Morales R, Soto C. Detection of prions in blood of cervids at the asymptomatic stage of chronic wasting disease. Sci Rep. 2017;7:17241. PubMed https://doi.org/10.1038/s41598-017-17090-x

31. Kramm C, Gomez-Gutierrez R, Soto C, Telling G, Nichols T, Morales R. In vitro detection of chronic wasting disease (CWD) prions in semen and reproductive tissues of white tailed deer bucks (*Odocoileus virginianus*). PLoS One. 2019;14:e0226560. PubMed https://doi.org/10.1371/journal.pone.0226560

32. Kramm C, Soto P, Nichols TA, Morales R. Chronic wasting disease (CWD) prion detection in blood from pre-symptomatic white-tailed deer harboring PRNP polymorphic variants. Sci Rep. 2020;10:19763. PubMed https://doi.org/10.1038/s41598-020-75681-7

33. Benavente R, Reed JH, Lockwood M, Morales R. PMCA screening of retropharyngeal lymph nodes in white-tailed deer and comparisons with ELISA and IHC. Sci Rep. 2023;13:20171. PubMed https://doi.org/10.1038/s41598-023-47105-9

34. Bravo-Risi F, Soto P, Benavente R, Nichols TA, Morales R. Dynamics of CWD prion detection in feces and blood from naturally infected white-tailed deer. Sci Rep. 2023;13:20170. PubMed https://doi.org/10.1038/s41598-023-46929-9

35. Sigurdson CJ, Williams ES, Miller MW, Spraker TR, O’Rourke KI, Hoover EA. Oral transmission and early lymphoid tropism of chronic wasting disease PrP^{res} in mule deer fawns (*Odocoileus hemionus*). J Gen Virol. 1999;80:2757–64. PubMed https://doi.org/10.1099/0022-1317-80-10-2757

36. Sigurdson CJ, Barillas-Mury C, Miller MW, Oesch B, van Keulen LJM, Langeveld JPM, et al. PrP^{CWD} lymphoid cell targets in early and advanced chronic wasting disease of mule deer. J Gen Virol. 2002;83:2617–28. PubMed https://doi.org/10.1099/0022-1317-83-10-2617

37. Spraker TR, Balachandran A, Zhuang D, O'Rourke KI. Variable patterns of distribution of PrP^{CWD} in the obex and cranial lymphoid tissues of Rocky Mountain elk (*Cervus elaphus nelsoni*) with subclinical chronic wasting disease. Vet Rec. 2004;155:295–302. PubMed https://doi.org/10.1136/vr.155.10.295

38. Saá P, Castilla J, Soto C. Ultra-efficient replication of infectious prions by automated protein misfolding cyclic amplification. J Biol Chem. 2006;281:35245–52. PubMed https://doi.org/10.1074/jbc.M603964200

39. Chen B, Morales R, Barria MA, Soto C. Estimating prion concentration in fluids and tissues by quantitative PMCA. Nat Methods. 2010;7:519–20. PubMed https://doi.org/10.1038/nmeth.1465

40. Sigurdson CJ, Aguzzi A. Chronic wasting disease. Biochim Biophys Acta. 2007;1772:610–8. PubMed https://doi.org/10.1016/j.bbadis.2006.10.010

41. Bartz JC, Marsh RF, McKenzie DI, Aiken JM. The host range of chronic wasting disease is altered on passage in ferrets. Virology. 1998;251:297–301. PubMed https://doi.org/10.1006/viro.1998.9427

42. Perrott MR, Sigurdson CJ, Mason GL, Hoover EA. Evidence for distinct chronic wasting disease (CWD) strains in experimental CWD in ferrets. J Gen Virol. 2012;93:212–21. PubMed https://doi.org/10.1099/vir.0.035006-0

43. Hamir AN, Kunkle RA, Cutlip RC, Miller JM, O'Rourke KI, Williams ES, et al. Experimental transmission of chronic wasting disease agent from mule deer to cattle by the intracerebral route. J Vet Diagn Invest. 2005;17:276–81. PubMed https://doi.org/10.1177/104063870501700313

44. Castilla J, Gonzalez-Romero D, Saá P, Morales R, De Castro J, Soto C. Crossing species barrier by PrP^{Sc} replication in vitro generates new infectious prions. Cell. 2008;134:757–68. PubMed https://doi.org/10.1016/j.cell.2008.07.030

45. Peden AH, Suleiman S, Barria MA. Understanding intra-species and inter-species prion conversion and zoonotic potential using protein misfolding cyclic amplification. Front Aging Neurosci. 2021;13:716452. PubMed https://doi.org/10.3389/fnagi.2021.716452

46. Herbst A, Velásquez CD, Triscott E, Aiken JM, McKenzie D. Chronic wasting disease prion strain emergence and host range expansion. Emerg Infect Dis. 2017;23:1598–600. PubMed https://doi.org/10.3201/eid2309.161474

47. Nichols TA, Fischer JW, Spraker TR, Kong Q, VerCauteren KC. CWD prions remain infectious after passage through the digestive system of coyotes (*Canis latrans*). Prion. 2015;9:367–75. PubMed https://doi.org/10.1080/19336896.2015.1086061

48. VerCauteren KC, Pilon JL, Nash PB, Phillips GE, Fischer JW. Prion remains infectious after passage through digestive system of American crows (*Corvus brachyrhynchos*). PLoS One. 2012;7:e45774. PubMed https://doi.org/10.1371/journal.pone.0045774

49. Baune C, Wolfe LL, Schott KC, Griffin KA, Hughson AG, Miller MW, et al. Reduction of chronic wasting disease prion seeding activity following digestion by mountain lions. MSphere. 2021;6:e0081221. PubMed https://doi.org/10.1128/msphere.00812-21

50. Kurz JC, Marchinton RL. Radiotelemetry studies of feral hogs in South Carolina. J Wildl Manage. 1972;36:1240. https://doi.org/10.2307/3799254

51. Singer FJ. Wild pig populations in the national parks. Environ Manage. 1981;5:263–70. https://doi.org/10.1007/BF01873285

52. Baber DW, Coblentz BE. Density, home range, habitat use, and reproduction in feral pigs on Santa Catalina Island. J Mammal. 1986;67:512–25. https://doi.org/10.2307/1381283

53. Boitani L, Mattei L, Nonis D, Corsi F. Spatial and activity patterns of wild boars in Tuscany, Italy. J Mammal. 1994;75:600–12. https://doi.org/10.2307/1382507

54. Cukor J, Linda R, Václavek P, Mahlerová K, Šatrán P, Havránek F. Confirmed cannibalism in wild boar and its possible role in African swine fever transmission. Transbound Emerg Dis. 2020;67:1068–73. PubMed https://doi.org/10.1111/tbed.13468

Appendix Table 1. Evaluation of prion infectivity contained in feral swine tissues using Tg1536 (cervid PrP expressing) mice*

Sample ID	Sample type	PrP^{Sc} signal/inoculated detection by western blot (%)	PrP^{Sc} signal/inoculated detection by PMCA (%)	Clinical signs/inoculated (%)	DPI
ID000000†	Brain	0/8 (0)	0/8 (0)	0/8 (0)	NA
ID0042049	Brain	0/11 (0%)	0/11 (0)	0/11 (0)	NA
ID0042049	SMLN	0/9 (0)	1/9 (11.1)	0/9 (0)	NA
ID0042059	Brain	0/8 (0)	0/8 (0)	0/8 (0)	NA
ID0042059	RPLN	0/9 (0)	0/9 (0)	0/9 (0)	NA
ID0042059	SMLN	0/6 (0)	0/6 (0)	0/6 (0)	NA
ID0042017	SMLN	0/7 (0)	4/7 (57.1)	0/7 (0)	NA
CWD Positive deer‡	Brain	8/8 (100)	8/8 (100)	8/8 (100)	359 $\pm$ 24.39
Pig-adapted PMCA§	PMCA product	In progress	In progress	In progress	TBD

*DPI, days post inoculation; ID, identification; NA, not applicable; PMCA, protein misfolding cyclic amplification; PrP^{sc}, disease-associated prion protein; RPLN, retropharyngeal; SMLN, submandibular; TBD, to be determined.
†Prion-free domestic pig.
‡Terminally ill, experimentally infected white-tailed deer.
§PMCA-adapted PrP^{Sc} from a scrapie-infected domestic pig. Adaptation performed in brain extracts from Tg002 mice (403 dpi as recorded on 07/30/2024).

Appendix Table 2. Evaluation of prion infectivity contained in feral swine tissues using Tg002 (porcine PrP expressing) mice*

Sample ID	Sample type	PrP^{Sc} signal/inoculated detection by western blot (%)	PrP^{Sc} signal/inoculated detection by PMCA (%)	Clinical signs/inoculated (%)	DPI
ID000000†	Brain	0/6 (0)	0/6 (0)	0/6 (0)	NA
ID0042049	Brain	0/8 (0)	0/8 (0)	0/8 (0)	NA
ID0042049	SMLN	0/6 (0)	0/6 (0)	0/6 (0)	NA
ID0042059	Brain	0/10 (0)	0/10 (0)	0/10 (0)	NA
ID0042059	RPLN	0/4 (0)	0/4 (0)	0/4 (0)	NA
ID0042017	SMLN	0/5 (0)	0/5 (0)	0/5 (0)	NA
ID0042059	SMLN	0/3 (0)	0/3 (0)	0/3 (0)	NA
CWD Positive deer‡	Brain	In progress	In progress	In progress	TBD
Pig-adapted PMCA§	PMCA product	In progress	In progress	In progress	TBD

*DPI, days post inoculation; ID, identification; NA, not applicable; PMCA, protein misfolding cyclic amplification; PrPsc, disease-associated prion protein; RPLN, retropharyngeal; SMLN, submandibular; TBD, to be determined.
†Prion-free domestic pig.
‡Terminally ill, experimentally infected white-tailed deer.
§PMCA-adapted PrP^{Sc} from a scrapie-infected domestic pig. Adaptation performed in brain extracts from Tg002 mice (376 dpi as recorded on 07/30/2024).

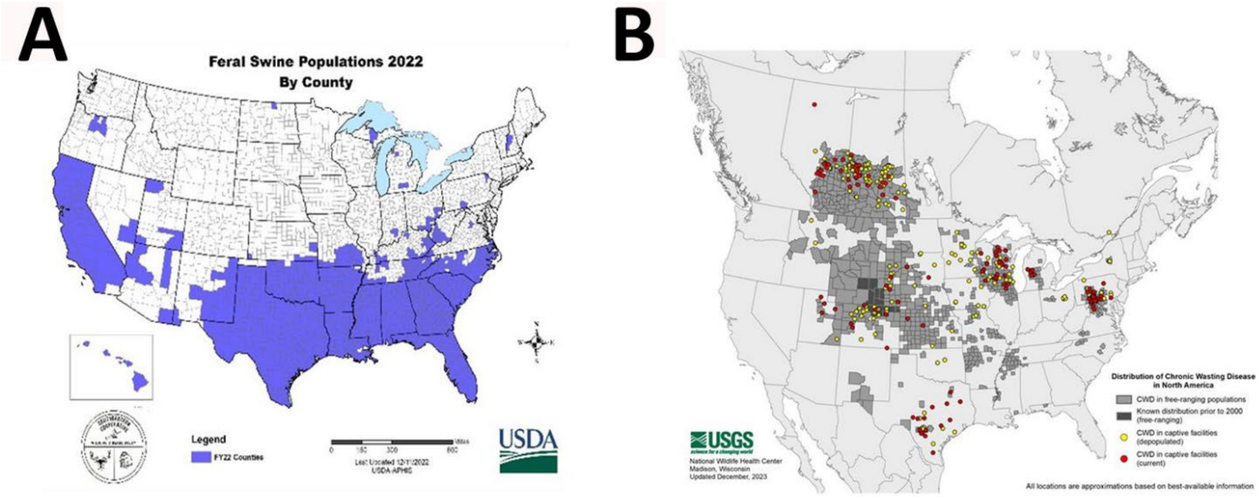

Appendix Figure 1. Geographic distribution of feral swine and CWD infected cervids in North America. A) The invaded range of feral swine across the United States (data obtained and prepared by USDA/APHIS). B) The distribution of chronic wasting in cervids in North America (data collected and prepared by USGS National Wildlife Health Center (Distribution of Chronic Wasting Disease in North America | U.S. Geological Survey (usgs.gov)). Panels (A) and (B) were reproduced with permission from USDA/APHIS and USGS National Wildlife Health Center, respectively.

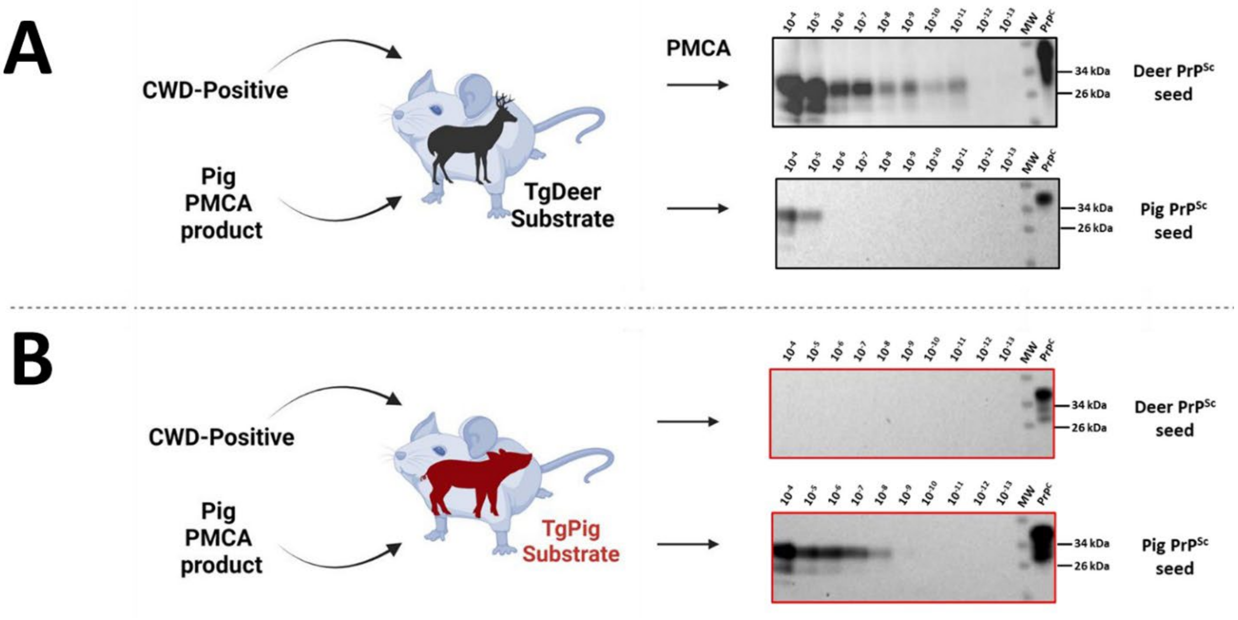

Appendix Figure 2. In vitro seeding activity of CWD and porcine-adapted prions using the PMCA technique. A) Evaluation of the prion replication activity of CWD and porcine-adapted prions in cervid PMCA substrate. B) Evaluation of the prion replication activity of CWD and porcine-adapted prions in porcine PMCA substrate. Each inoculum was tested at different dilutions (ranging from 10^{-4} to 10^{-13}) in a single PMCA round. Left panels in (A) and (B) depict experimental strategies. All PMCA products were PK digested before western blotting as described in Methods. PrP^{C} represents undigested (no-PK treatment) PrP substrates used as controls of electrophoretic mobility and antibody reactivity. Numbers at the left of the membranes represent molecular weight markers (MW, in KDa).

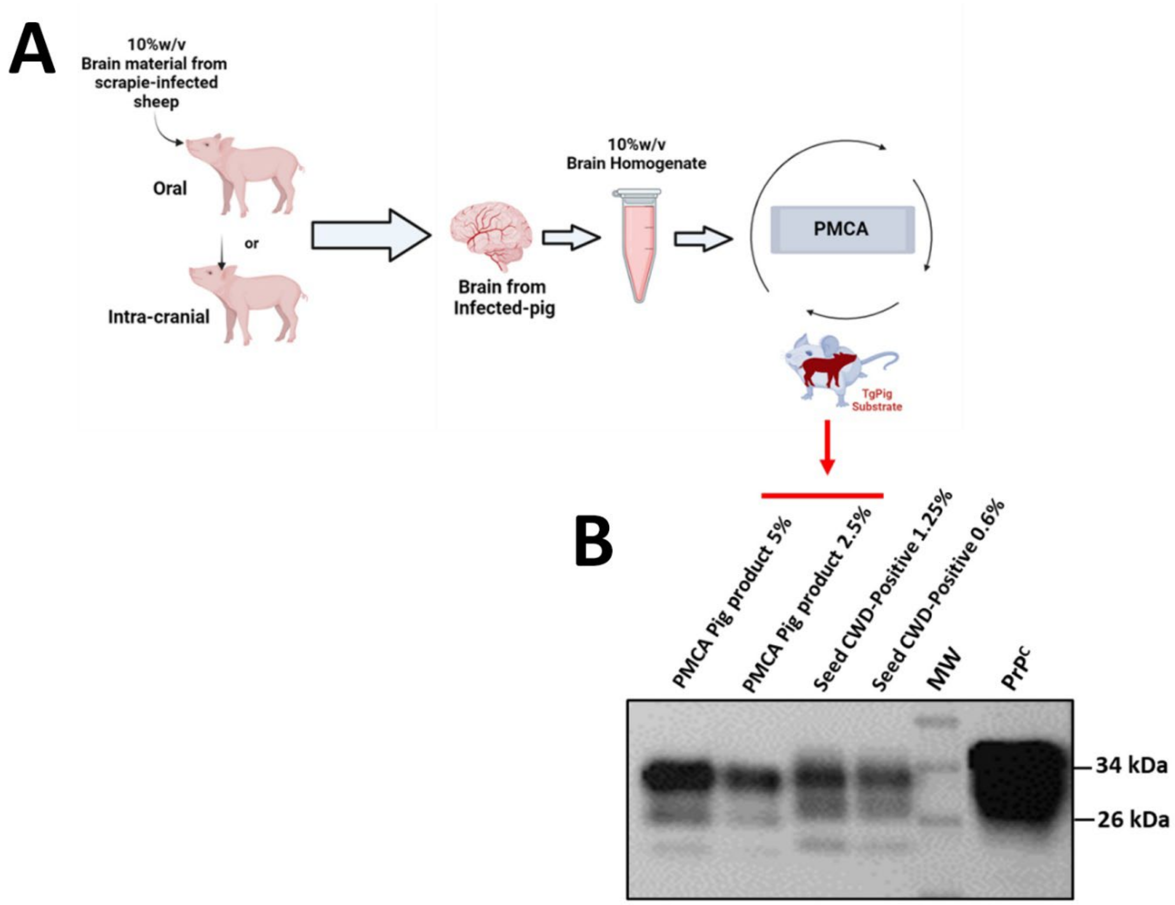

Appendix Figure 3. Characterization of the porcine adapted PrP^{Sc} seeds used in this study. A) Experimental strategy depicting the origin of the pig-adapted scrapie prions used to seed the PMCA reactions. This PrP^{Sc} material was obtained by serial PMCA passages (>10). B) Western blot of the porcine-adapted PMCA product (PMCA Pig product, noted by a red line) used as positive control in the porcine-PMCA reactions. This sample was visualized after serial dilutions (5% and 2.5%) and compared with CWD prions from a terminally ill, experimentally infected white-tailed deer (Seed CWD-Positive). These samples were PK-treated before western blotting as described in Methods. PrP^{C} represents undigested (no-PK treatment) PrP substrates used as controls of electrophoretic mobility and antibody reactivity. Numbers at the left of the membranes represent molecular weight markers (MW, in KDa).

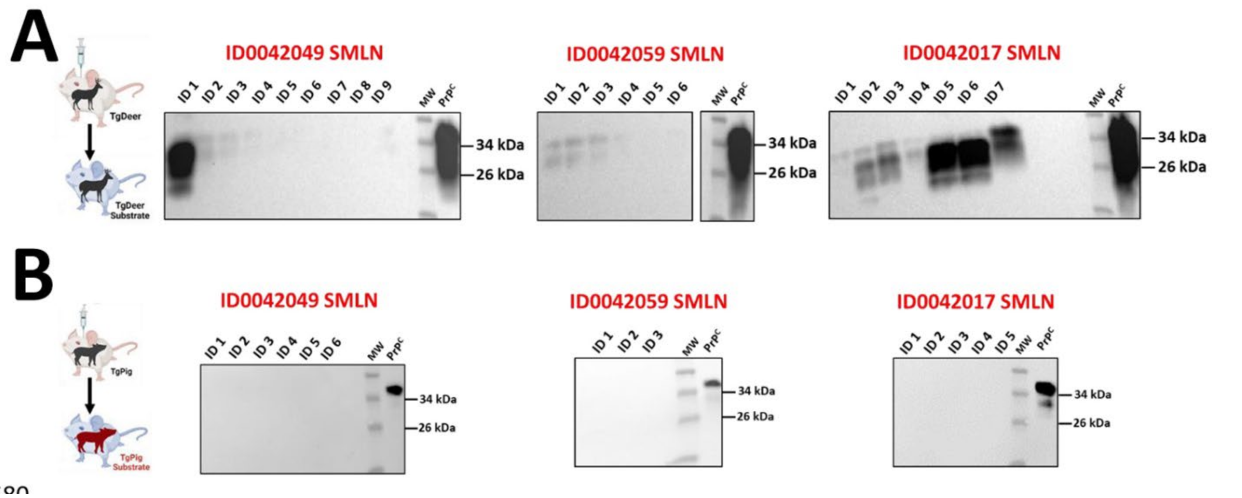

Appendix Figure 4. Representative PMCA reactions for mice's brains challenged with wild pig tissues. A) PMCA products of brains from Tg1536 mice inoculated with wild pig tissues. B) PMCA products of brains from Tg002 mice inoculated with wild pig tissues. PMCA reactions in each case were performed in a substrate/inoculum species homologous fashion as illustrated at the left side of each panel. All PMCA products correspond to a third PMCA round. These samples were PK-treated before western blotting as described in Methods. PrP^C represents undigested (no-PK treatment) PrP substrates used as controls of electrophoretic mobility and antibody reactivity. Numbers at the left of the membranes represent molecular weight markers (MW, in KDa).
